# Evaluating the socioeconomic benefits of heat-health warning systems

**DOI:** 10.1093/eurpub/ckae203

**Published:** 2025-02-09

**Authors:** Shilpa Rao, Prayash Chaudhary, Isabelle Budin-Ljøsne, Susan Sitoula, Kristin Aunan, Matthew Chersich, Francesca de’ Donato, Aleksandra Kazmierczak

**Affiliations:** Division for Climate and Environmental Health, Norwegian Institute of Public Health, Oslo, Norway; Department of Addiction Medicine, Norwegian Research Center for Agonist Treatment of Substance Use Disorders (NORCATS) and Bergen Addiction Research (BAR), Haukeland University Hospital, Bergen, Norway; Department of Global Public Health and Primary Care, University of Bergen, Bergen, Norway; Division for Climate and Environmental Health, Norwegian Institute of Public Health, Oslo, Norway; Department of Health Sciences, Norwegian University of Science and Technology (NTNU), Gjøvik, Norway; CICERO Centre for International Climate Research, Oslo, Norway; Wits Planetary Health Research Division, Faculty of Health Sciences, University of the Witwatersrand, Johannesburg, South Africa; Public Health and Primary Care, School of Medicine, Trinity College Dublin, Dublin, Ireland; Department of Epidemiology, Lazio Regional Health Service—ASL Roma 1, Rome, Italy; European Environment Agency (EEA), Copenhagen, Denmark

## Abstract

Heat-health early warning systems (HHWS) are an important collaborative activity between the meteorological and health communities. This study aimed to map the evidence on the socioeconomic assessment of HHWS and their effectiveness in terms of averting heat related health outcomes. It also aimed to map the technical, structural, and societal barriers and facilitators to implementation and use of HHWS. We use two methods: (i) a scoping review of literature on the economic assessment and health benefit of climate services for heat-health adaptation (ii) a set of interviews with climate service developers and providers in Europe and Africa to understand further technical and societal aspects as well as evaluation of such services. We find that HHWS can be a cost-effective adaptation option that can reduce heat-related mortality and morbidity, especially in vulnerable groups like the elderly. We find that challenges such as lack of long-term and reliable funding, difficulties in making the climate data relevant, comprehensible, and accessible to different end-users, cultural differences between climate and health professionals, and limited ability to assess the services’ real impact need to be accounted for while implementing these services.

## Introduction

Heat-health warning systems (HHWS) are climate services used worldwide to provide early notification to decision-makers and the public about hazardous heat situations and heat waves [1]. HHWS usually combine weather forecasts with health data to provide ‘graded’ alert systems that can be used to initiate immediate public health interventions aimed at minimizing the adverse impacts of heat stress on human well-being during extreme heat conditions. The majority of HHWS are established and managed by National Meteorological and Hydrological Services in collaboration with health services managing national or regional heat health action plans (HHHAPs) [2, 3].

There is a recent focus on evaluating the socioeconomic advantages of early warning systems and support the United Nations Secretary General’s Early Warnings for All initiative [4]. Recent studies have highlighted the need for developing smart population-health-oriented early warning systems with full coverage of heat-health risk management, which allow identification of automatic warning signals on the basis of characteristics of the health concerns due to heat [5]. However, only 18% of the 193 World Meteorological Organization (WMO) Member States reported conducting socioeconomic benefit assessments of early warning systems in the last 10 years [6]. The evaluation of such services is compounded by the challenges of the unavailability of sufficient data on the benefits and a lack of information on the societal aspects of these services [7].

In this study, we first used a scoping review to analyse the current evidence on the economic assessment and health benefit of HHWS and second conducted an interview analysis with selected parties in Europe and Africa to examine if the needs, risk perception, behaviour, and practices of targeted users are integrated in the design of the services. This study was conducted as part of the recently concluded Horizon 2020 project ENBEL (Enhancing Belmont Research Action to support EU policy making on climate change and health) that specifically focussed on research in Europe and Africa.

## Methods

We focus on the following research questions:

1. What are the costs and benefits of HHWS?
2. How do HHWS impact mortality, morbidity, and healthcare services?
3. Are the needs, risk perception, behaviour, and practices of targeted users integrated in the design of HHWS services?

We conducted the study with two parts- a scoping review and an interview-based analysis in Europe and Africa.

### Scoping review

We used the Joanna Briggs Institute methodology for the scoping review [8]. We developed a protocol but did not publish it.

We developed the research question for the review using the Population or problem, Concept, and Context (PCC) framework which is summarized along with the inclusion and exclusion criteria in Table 1. The scoping review included peer-reviewed journal articles as well as grey literature and was conducted from December 2022 to April 2023.No restriction for the year of publication or geographical area was applied. We searched Medline (PubMed), Embase (Ovid) and Web of Science databases for the journal articles as well as grey literature from WHO Institutional Repository for Information Sharing, the WMO website, the Climate-ADAPT website, and Google Scholar (see Supplementary Information).

**Table 1.** Overview of PCC framework along with the inclusion and exclusion criteria

Inclusion criteria	
General criteria	Peer-reviewed journal articles (primary studies) and reports The article is in English No restriction for the year of publication or geographical area
Participants	Literature presenting climate services targeted toward the general population or any specific vulnerable group such as outdoor workers or the elderly. Humans and not animals or plants
Concept	Literature presenting economic assessment (Cost-benefit analysis, Cost-Effectiveness Analysis, Multi-criteria analysis) Cost parameters could include the operational cost of climate services such as the cost of triggering heat warnings as well as the cost of response measures such as the cost of disseminating climate information to the public or the cost of healthcare professionals personally taking care of vulnerable people following heat warnings. Literature presenting evaluation or use of climate services for heat-health adaptation in reducing health impacts (mortality, morbidity) or changes in the use of healthcare services such as emergency department visits.
Context	Services must be targeted to adapt to health risks posed by extreme heat events. Services for health could be in the form of a heat-health/early warning system/app/tool, weather forecasting system/model/application, alert system

Exclusion criteria	

	Absence of full text, review articles, a study may present an example or description of operational climate services for health but does not present an economic assessment or direct or indirect evaluation of its effect on health outcomes, climate services towards extreme weather event response planning and support other than heat events such as flood, storm, or drought.

**Figure 1.**
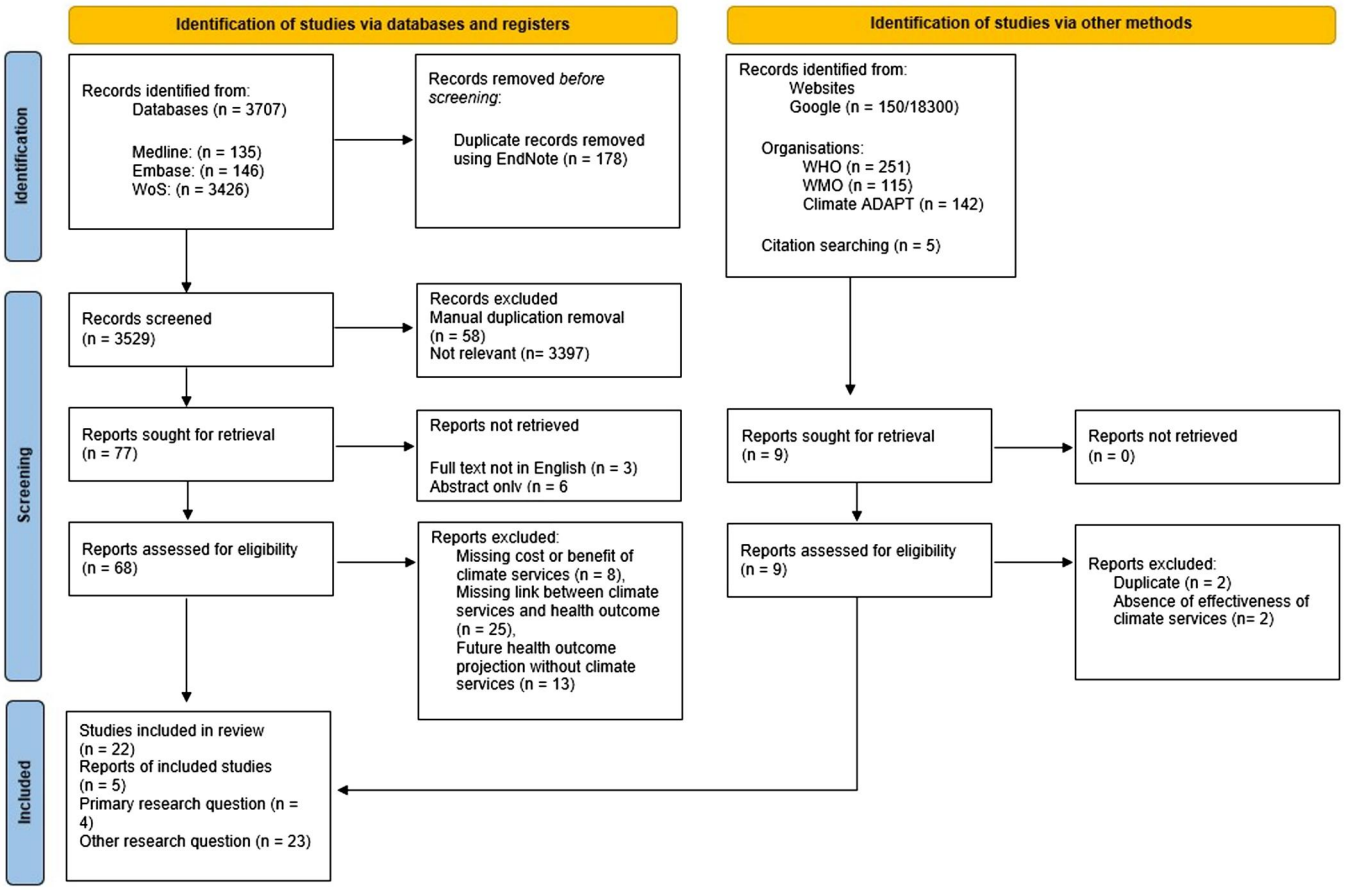
PRISMA-ScR flowchart for evidence screening and selection process.

We provide detailed information on search strategy, search terms, selection of evidence and data extraction in the Supplementary Information (SI). The results of the search and the study inclusion process are presented in the PRISMA-ScR flow diagram in Fig. 1.

### Interviews

We invited seven representatives involved in the development of HHWS in Europe and Africa and four representatives from two public health institutions in Europe (France and the UK) to join an online semi-structured interview, using a convenience sampling approach. Interviews were conducted between February and December 2023. We developed an interview guide comprising 10 questions to investigate issues related to stakeholder engagement in the development of the services, funding, capacity-building, communication, service evaluation, and barriers encountered in the development and implementation of the services. Two researchers from the Norwegian Institute of Public Health moderated the interviews using Microsoft Teams. The interviews lasted 40 minutes on average, were video recorded, transcribed verbatim, and we analysed these in Microsoft Word using a simple inductive content analysis approach [9] (see Supplementary Information).

## Results

### Scoping review

We obtained a total of 3707 records from all three databases and a total of 27 studies were selected for this review.

Of these 27 studies, four studies presented an economic assessment and the other 23 studies reported changes in health outcomes or changes in healthcare services utilization after heat warning or implementation of heat warning systems and other public health measures mostly defined in a HHHAP. While 16 studies measured health outcomes in terms of mortality, four studies measured morbidity and three studies measured both. Other typical characteristics of the studies in terms of geographical location, sample population, and study design or analytical methods used are in Table 2.

**Table 2.** General characteristics of studies included in the scoping review

Economic assessment							
Authors (publication year)	Type of CS used	Study period	Country/Region	Sample size/population studied	Study design OR Methods used	Outcome (Health outcomes measured)	Methods used for estimating monetary value of health outcomes
	HHWS	2020–40	Madrid, Spain	General Population	Economic evaluation (cost-benefit analysis)	Benefit-cost ratio (Mortality)	VSL, VOLY
[8]	HHWS	1995–98	Philadelphia, USA	Considered mortality reductions among >65 years old	Economic evaluation (cost-benefit analysis)	Net benefit (Mortality)	VSL
[9]	HHWS	2015–64	London, England Madrid, Spain Prague, Czech Republic	General population	Economic evaluation (cost-benefit analysis)	Benefit-cost ratio (Mortality and patient day in hospital)	VPF, publicly available information on health care service use
[10]	HHWS	2009 (pre-intervention) and 2014 (post-intervention)	Adelaide, Australia	General Population	Economic evaluation (Cost-Benefit Analysis)	Benefit-cost ratio (Morbidity, ambulance call outs, heat related admission, emergency department presentation)	Publicly available information on health care service use

**Health Outcome Assessment**							
**Authors, publication year**	**Type of CS used**	**Study period**	**Intervention or focus of evaluation/study**	**Region, Country**	**Sample population/subgroups**	**Study design or Methods used**	**Outcome measured**
Studies evaluating effectiveness of heat warning/alert/notification							

[11]	Heat Warning	1997–2005	Very Hot Weather Warning	Hong Kong	Elderly (65 years or older)	Observational study (Multiple Linear Regression)	Mortality due to IHD and stroke
[12]	Heat Early Warning and Advisory	2015	Téléphone Santé, automated phone warning and advisory system	Montreal, Canada	At least one of the following: 65 years of age or older, present a heart or lung medical condition, suffer from diabetes, kidney failure or a neurological disorder, or, lastly, have mental health issues	Randomized controlled study	Health services use, Morbidity.
[13]	Heat Early Warning and Advisory	2006–16	Heat warnings and heat advisories	2800 counties, USA	Elderly (aged 65 and older)	Conditional Poisson regression models	Mortality and Morbidity (Hospital admission)
[14]	Heat EW and Advisory	2001–06	Heat warnings and heat advisories	20 cities, USA	General population	Case-crossover design; conditional logistic regression	Mortality rate

Studies reporting changes in health outcomes/healthcare service utilization after implementation of HHWS as a part of wider HHAP or HHWS with emergency response measures							

[15]	HHWS	2006–07 (before change in threshold) 2009–10 (after change in threshold)	Threshold change of heat emergency plan	New York City, USA	elderly (65 years or older)	Quasi-experimental (Difference-in-Differences method coupled with propensity-score matching)	Morbidity
[16]	HHWS	May to September from 2012–16 (pre intervention) 2016–18 (post intervention)	Harmonized Heat Warning and Information System along with emergency public measures followed by warning	Ontario, Canada	General population, also examined effect on heat-vulnerable groups (≥65 years or <18 years, those with comorbidities, those with a recent history of homelessness)	Quasi-Experimental (interrupted time series analysis)	Emergency department visits for heat-related illness
[17]	HHWS	1999–2002 (pre-intervention) 2005–08, 2009–12,2013–16 (post- intervention)	HHWS and National heat plan	23 cities, Italy	Aged 65 and over	Time series Analysis, Distributed lag non-linear model	Mortality
[18]	HHWS	1975–2003 (pre- intervention) 2004–06 (post- intervention)	HHWS and National heat wave plan	France	General Population	Generalized Estimating Equations with a Poisson distribution	Mortality
[19]	HHWS	2009–14	Heat warning system and emergency measures following warning	Seven cities, Korea	General Population; (young <18, elderly 65+, different sex, education attainment, marital status, job status)	Quasi Experimental (difference-in-differences)	Mortality (Risk of mortality, all-cause mortality, and CVD & respiratory mortality)
[20]	HHWS	2007–10 (pre-intervention) 2014–15 (post-intervention)	Heat warning system and HAP	Ahmedabad, India	General population	Distributed lag nonlinear model	Mortality
[21]	HHWS	2003–13 (2006–13 post-intervention)	Heat warning system and HHAP	Frankfurt am Main, Germany	General population; Elderly (60+)	Kruskal-Wallis test and Mann Whitney test	Mortality
[22]	HHWS	1993–2002 (pre- intervention) 2004–13 (post-intervention)	Heat alert system and regional Heat Health Prevention Plans	Spain	General Population Elderly (65+), different sex,	Time-varying distributed lag non-linear models	Mortality
Martinez-Solanas and Basagana (2019 b)	HHWS	1997–2002 (pre- intervention) 2004–13 (post- intervention)	Heat alert system and Heat Health Prevention Plans	Spain	General population; Elderly (65+), different sex,	Distributed lag non-linear models	Morbidity (hospital admission)
[23]	HHWS	1995–2002 (pre-intervention) 2003 and 2004 (post-intervention)	HHWS and prevention programs targeting elderly and other high-risk subgroups	Four cities, Italy	General population	Segmented regression and generalized additive models	Mortality
[24]	HHWS	1999–2002 (pre- intervention 2004–07 (post- intervention)	Regional heat warning system	Florentine area (Tuscany), Italy	older adults (aged 65–74) and in elderly ≥75 years old	Case-crossover time-stratified analysis	Mortality
[25]	HHWS	2009 (pre- intervention) 2014 (post- intervention)	Heat warning and adaptive public health measures following the warning	Adelaide, Australia	General Population; (younger adults <18, elderly +65)	Case-series analysis	Mortality and Morbidity (Hospital admissions)
[26]	HHWS	1995 (pre-intervention) 1999 (post-intervention)	Hot Weather Health Warning and Advisory	Chicago & St. Louis, USA	General population	Retrospective observational study	Mortality
[27]	HHWS	1995–2002 (pre- intervention) 2004–13 (post- intervention)	Early heatwave alerts for health professionals and intensified care of susceptible individuals	Eight cities, Switzerland	General population, Different sex, elderly (74+)	Conditional quasi-Poisson regression models with non-linear distributed lag functions	Mortality
[28]	HHWS	1998–2002 (pre-intervention) 2006–10 (post-intervention)	HHWS and HHAP	16 cities, Italy	Elderly over 65 years of age	Generalized Additive Model and multivariate meta-analysis	Mortality
[29]	HHWS	2003 (pre-intervention) 2006, 2010, 2015 (post-intervention)	HHWS and HAP	Hesse, Germany	General population; elderly (60+)	Time series regression	Mortality
[30]	HHWS	1998 (pre intervention) 2003 (post intervention)	HHWS along with other socioeconomic factors	Shanghai, China	General population. Socioeconomic factors, elderly 65+	Multivariant regression analysis	Mortality
[31]	HHWS	1995 vs 1999	Heat advisory and emergency response measures following the warning	Milwaukee, USA	General population, Elderly 65+, different sex, socioeconomic status, race	Poisson regression analysis	Mortality and Morbidity

VSL, value of statistical life; VOLY, value of a life year; VPF, value of a prevented fatality.

#### Economic assessment

We included four studies [10, 18, 27, 28] that assessed the cost and benefits of HHWS. The key findings from the four studies concluded HHWS to be a ‘no/low regret adaptation’ producing a benefit-cost ratio greater than 1 in current and some future climate change scenarios. All four studies quantified the monetary value of reduced heat-related mortality and the reduced healthcare utilization after the implementation of HHWS. Different methods such as value of statistical life, value of a life year, value of a prevented fatality and use of publicly available information on health service utilization were used to quantify the health benefits. The studies further categorized the cost of HHWS into two groups: operational cost of running the warning system and additional cost associated with health action plan. The operational cost per heatwave day was estimated at 7800 Euros, while including the additional cost of heat action plan, the cost varied between 9261 Euros and 14 000 Euros (conversion rate of 1 US Dollars = 0.9261 Euros, March 2023 value) [10]. Another study reported an additional fixed annual cost of contract fee with the weather office and warning dissemination costs at 200 000 Euros [28]. Regarding costs of HHWS, we only found one study that estimated costs at 371 811 Euros (conversion rate of 1 Australian Dollars = 0.6270 Euros, March 2023 value) for implementing the HHWS and targeted intervention for 7 days [17].

#### Health outcomes and use of HHWS

There are 23 peer-reviewed studies (see Table 2) that investigated changes in heat-related mortality and morbidity after heat wave alert or implementation of heat warning systems and other public health measures mostly defined in a HHAP. One study used an experimental study design [21]; two studies used the difference-in-differences method [29]; and one study used interrupted time-series analysis [24] to estimate the causal effect of heat alert or heat warning system following the warning on the health outcomes. The remaining 19 studies used other time series, regression analysis, or non-parametric tests mostly in a pre/post-intervention study period to establish an association between the heat warning system and health outcomes or an association between temperature and mortality in two time periods to attribute the changes in temperature-mortality relationship to the implementation of HHWS and HHAP.

In Europe, several studies estimated heat-related deaths averted post-HHAP implementation in Italy, Germany, France, Spain, and Switzerland with expected deaths averted ranging from 2% to 23% [11, 15, 19, 20, 26, 30]. Some studies [15, 26] found inconclusive evidence on the effectiveness of the plan due to varying heatwave characteristics. There were also studies that showed more impacts in vulnerable populations [20, 22, 26]. In Asia, reductions in health effects were observed in India, Shanghai, and Hong Kong [12, 16, 32], with all or part of these being attributed to HHWS. Conversely, one study found increased mortality among the elderly and cardiovascular mortality [14]. In the USA and Canada, [31] found effective heat emergency response systems in Chicago and St Louis; while [29] found greater benefits for the elderly and low socioeconomic neighbourhoods in Montreal, Quebec; while [25] found varying impacts of heat alerts on mortality across US cities, with significant reductions only in Philadelphia.

For morbidity, a significant reduction in heat-related illnesses per day was observed in New York city and in Spain post-implementation of heat prevention plan following a heat warning [13, 29]. Again, some studies found no or inconclusive evidence [24], with some indicating more impacts among women, chronically ill and socioeconomically disadvantaged populations [21, 24]. Some studies estimated both mortality and morbidity and found fewer ambulance callouts, hospital admissions, and emergency presentations during heatwaves after implementing a heat warning system and increased hospitalizations for heat stroke and fluid/electrolyte disorders [17, 25, 33].

There were also mixed results in terms of the use of health services and some studies did not find an increase in people who assessed the healthcare system, or for any subgroups [17]. Some studies found that individuals with a recent history of homelessness had an increase in the rate of emergency room visits [17, 24].

### Interviews

The following text summarizes the main input from the interviews.

#### About the HHWS

Most HHWS were developed as collaborations between climate experts and public health authorities and funded by the European Commission through time-limited projects or through national administrations and ministries. The HHWS were often integrated parts of national HHHAPs. Two HHWS in our sample were specifically designed to be used by outdoor workers and managers within sectors such as manufacturing, construction, transportation, agriculture, as well as health care, and these HHWS involved the users in product development by organizing meetings, workshops, and surveys in different countries. To ensure broad use, the HHWS offered capacity-building to target users such as training sessions with outdoor workers or health care staff to learn how to interpret early warning information, recognize heat-related dehydration, and mitigate heat stress. Other training tools included infographics, posters, videos, or action cards to recommend specific actions to social and healthcare workers. Information about the tools was disseminated to the public through websites, apps, social media, national helplines, and the organization of workshops and conferences.

#### Challenges in the development and implementation of HHWS

The HHWS representatives encountered several challenges in the development and implementation of the HHWS as described below. Specific comments from the interviewees are described in Supplementary Table S24.

#### Data collection and reliability

The representatives explained that developing reliable heat warnings and weather forecasts can be challenging for several reasons. The data needed to feed the models may not be available in time or be too expensive to access. In such cases, other data might have to be used, that may have limited relevance for Europe. One representative explained that delivering heat warnings in time is demanding given the types of data that must be collected and processed. Combining climate and health data to produce meaningful warnings, was seen as highly complex by the interviewees because for example, metabolic characteristics differ from one individual to another and collecting data about people’s habits and health may also be hindered by privacy protection norms.

#### Interdisciplinary work

The representatives explained that collaboration between the health and the climate sector can be hampered because of the use of different jargons and data. Climate professionals and health professionals have diverse ways to collect, analyse and understand temperature data for example. Using different ground temperatures may be more useful for a climate professional and less for a health professional. Another representative explained that health data are more sensitive than climate data, which may be difficult to understand for professionals who are not used to follow stringent privacy and confidentiality requirements. Although collaboration between the climate and health sector has been strengthened in recent years, there is still an issue of overlap, and responsibility in dealing with HHWS. The representatives however still agreed that interdisciplinary collaboration was a useful learning and practical experience.

#### Awareness raising and communication

The representatives found it difficult to know whether their institutional stakeholders used the HHWS forecasts. They experienced that interest in such forecasts is high in times of extreme heat, less so when the weather is moderate. It was time consuming for them to keep track of whom to inform in public administration. The representatives also experienced that populations living in remote areas or in areas of low socioeconomic status are less interested in following heat warnings. This could be because they have more urgent priorities, such as securing access to basic infrastructure. Some groups, e.g. athletes, also do not consider themselves at risk and others like health staff are not sufficiently aware of heat related risks. The HHWS representatives found it resource-demanding to ensure that the HHWS are used, are user-friendly and provide understandable, multilingual and practical guidance to end-users. The representatives also explored the use of existing communication channels, for instance by integrating climate information like wind chill index or heat stress index in for e.g., Google platform. Providing information at local shops or through TV channels and pamphlets, as also seen as a potential strategy to spread information at low cost.

#### Funding

Most representatives experienced that the funding of their HHWS was not sustainable. Project-based services could not be pursued due to loss of funding after project completion. Although some HHWS benefit from funding through national administrations and ministries that is not time-limited, their representatives experienced that such funding was reduced over the years despite the need to ensure maintenance and development. Interviewees also complained on the lack of prioritization on collecting data to document the impact of heat on health. The representatives also reported a lack of a clear direction on funding and means to properly communicate information, realize HHHAPs or conduct basic activities to inform about heat and health.

#### Impact

The representatives found it challenging to evaluate the impact of the HHWS. Quantifying impact, for instance the number of service users, was seen as hardly feasible as the HHWS do not have a full overview of which groups are the end-users. For instance, smartphone applications can be used without having to register, thus the number of users remains unknown. Some HHWS use models to calculate reductions in mortality. It is, however, difficult to know whether such reductions are due to the use of the HHWS or are the result of diverse actions conducted by the authorities, such as information and prevention campaigns. Assessing impact in time-limited projects is also challenging if required within the project’s limited period. Despite the lack of solid evidence, many representatives believed that HHWS has lead to some positive impacts in society including for example changes to work practices for outdoor workers.

A summary of all relevant aspects related to the interviews is in Table 3.

**Table 3.** Challenges encountered in the development and implementation of HHWS

Data collection, reliability, and sharing
Services need data that may not be available free of charge, e.g. weather data Available data may not be relevant in the context of the service, e.g. if no local weather data available Uncertainties remain regarding the reliability of some data, e.g. mosquito spreading data Demanding to collect the data necessary in due time to produce heat warnings Difficult to produce simple and understandable data models Challenging to produce personalized information about heat health as individual characteristics vary, e.g. ability to tackle dehydration Challenging to obtain data about people’s habits and health due to privacy concerns
**Interdisciplinarity**
Communication between professionals from different disciplines can be challenging due to the use of different jargons Difficult for climate professionals to evaluate the health impact of climate data on health
**Awareness raising, communication, and dissemination**
Interest among stakeholders for heat warnings may be difficult to maintain over time if the weather is moderate Some groups may be difficult to reach if they do not consider themselves vulnerable to heat/cold, e.g. athletes Health care staff may not be aware of the risks of heat or climate change issues, e.g. heat-related dehydration Challenging to produce user-friendly, understandable information, also in multiple languages Demanding to accompany warnings with practical guidance regarding what to do in case of extreme weather events Demanding to maintain contacts with relevant stakeholders, also if located in geographically remote areas
**Funding**
Lack of long-term and reliable funding to maintain services over time Lack of evidence to document the need for funding climate services, e.g. mortality data Insufficient funding to communicate heat warnings and realize HAPs

## Discussion

The findings demonstrate that HHWS is a crucial part of a HHAP and along with other public health actions, following a warning can help in reducing heat-related mortality and morbidity, especially among some subpopulations.

Most of these studies we reviewed were conducted in the European countries, the USA, and Canada, highlighting gaps in studies from South American, African and many Asian countries. We see a positive trend in more economic evaluations on HHWS being conducted in recent years than in the past (see Supplementary Information). The reviewed studies showed mixed results with indications of correlation and not causation in the results. There is a need for future studies which try to disentangle the effectiveness of individual preventive measures within HHAP and evaluate the effectiveness of a HHWS independently and use experimental and quasi-experimental study designs to account for the confounding factors. The studies included in the review also highlighted the need to consider local epidemiological evidence and include regional variations like the awareness of health risks associated with heat, individual preventive measures such as proper hydration and use of air conditioning, shifting population demographics, air pollution and changes in living conditions which contribute to a reduction in heat-related health outcomes.

The key findings from the four studies concluded HHWS to be a ‘no/low regret adaptation’ producing a benefit-cost ratio greater than 1 in current and some future climate change scenarios. The uncertainty in the cost-effectiveness of HHWS in future climate scenarios is associated with, among other things, the methods to value health risks or benefits; applied discount rate in measuring future costs; health sensitivity to ambient temperature; and the life span of a HHWS.

A HHWS is usually implemented as a part of a wider HHAP. The absence of a designated budget for the heat action planning process, limited data on the number of personnel and time dedicated per type of action, and difficulty in allocating the specific cost of assessing weather and climate information from meteorological agencies, are some of the challenges faced in quantifying the true cost of a HHWS. We see a need for studies with a comprehensive and transparent analysis of resource costs. Furthermore, we find that all these costs were calculated assuming that the most expensive components, such as earth observation satellites necessary to provide weather forecasts, are already in place.

Another key uncertainty highlighted in the economic assessment studies was the methods to value the health risks and benefits. We note a lack of specific alternative metrics to value mortality in the context of heat waves given that cost-effectiveness decisions can vary significantly depending on the valuation method. These studies also suggested that the monetary benefit of reduced morbidity is lower than that associated with reduced mortality thus also highlighting the need for improved valuation methods to validate these results.

In this evaluation, we focussed on health benefits related to reduced mortality, morbidity, and healthcare service use. Most studies focussed on mortality as a health outcome measure, with few studies on morbidity and healthcare service only coming out in recent years (see Supplementary Fig. S2). We did not account for additional health benefits such as improved health behaviour and increased awareness of risk associated with extreme heat. Furthermore, the climate services for health were restricted in the context of heat waves and heat adaptation. Inclusion of a wider range of parameters could have an impact on our results.

The selection of studies we reviewed was limited to the English language. As a result, articles which were deemed suitable during the title and abstract screening process had to be excluded as the full-text articles were in languages other than English. This further limited the inclusion of evaluation documents which are published on governmental websites and were not in English.

The HHWS representatives we interviewed in Europe and Africa indicated that sustaining interest in heat alarms and climate information was challenging. Fostering collaborations with relevant and trusted stakeholders may contribute positively to maintain interest. Using multiple communication channels and providing messages that are culturally sensitive, easily understandable, and available in the languages spoken by the target groups, may be efficient and is in line with recent recommendations by public health agencies. Engaging vulnerable groups in the design and development of HHWS, and incorporating their perspectives to ensure the services meet their needs, for instance by organizing citizen assemblies to ensure the inclusive design of climate services, may also be important [34].

Climate information often involves scientific terminology and uncertainty, making it difficult for non-experts to comprehend and use effectively, thus often leading to misinterpretation. Encouraging partnerships between scientists from different disciplines and the intended users of the services may be useful to ensure that the services better align with the users’ needs [23]. Ensuring effective communication, including the use of multiple languages and culturally appropriate messaging, is essential to ensure equitable access to climate services, particularly for vulnerable populations.

We interviewed a limited sample of representatives that cannot be considered representative of climate services and public health in general. Our findings may, therefore, not suffice to draw robust conclusions. A major constraint was the inclusion of only one stakeholder for Africa in the interview. However, there were no significant discrepancies in the feedback we received from the interviewees geographically, thus suggesting that these inputs may provide some useful insight regarding the overall societal impact of climate services.

To conclude, fostering collaborations between climate service providers, researchers, decision-makers, enhancing the accessibility, and availability of climate data and information, and investing in capacity building and training programs, can contribute to making HHWS an effective part of heat health action plans.

## Supplementary Material

ckae203_Supplementary_Data

## Data Availability

The data underlying this article are available in the article and in its online supplementary material.
Key points Heat Health warnings systems can be a cost-effective adaptation option that could reduce mortality, morbidity, and use of health care services among the public. They are particularly useful to subpopulations like the elderly. We find a gap in studies evaluating the cost-effectiveness of HHWS due to uncertainties in methods to value health risks/benefits, the effectiveness of HHWS, the cost estimate of the warning system, health sensitivity to hot ambient temperature, the life span of a HHWS and climate sensitivity. Current HHWS face challenges of data availability and reliability, interdisciplinarity, outreach and budget constraints. Fostering collaborations between climate service providers, researchers, decision-makers, enhancing the accessibility and availability of climate data and information, and investing in capacity building and training programs, can contribute to making HHWS more efficient and impactful in the future endeavors. These findings can be useful to the EU in their plans to integrate HHWS into broader adaptation strategies and ensure that such services are cost-effective and involve all parts of society in their outreach.
