# Supplementary material for: Evaluating the socioeconomic benefits of heat-health warning systems
Source: Eur J Public Health. 2025 Feb 9;35(1):178–86. doi: 10.1093/eurpub/ckae203 (PMC11832133; doi:10.1093/eurpub/ckae203)
Supplement: ckae203_Supplementary_Data [file ckae203_supplementary_data.zip › ckae203_Supplementary_Data/ejph-2024-05-om-0342-File006.docx]

**SUPPLEMENTARY MATERIAL**

## S1: Scoping review methodology and search string

***Inclusion Criteria***

Peer-reviewed journal articles (primary studies) and grey literature presenting the economic assessments (Cost-benefit analysis, Cost-Effectiveness Analysis, Multi-criteria analysis) of climate services for health targeting the general population or any specific vulnerable group. The services could include HHWS, a digital application or tool, a weather forecasting system/model/application, or an alert system. Cost parameters could include the operational cost of climate services such as the cost of triggering heat warnings as well as the cost of response measures such as the cost of disseminating climate information to the public or the cost of healthcare professionals personally taking care of vulnerable people following heat warnings. Literature presenting evaluation or use of climate services for heat-health adaptation in reducing health impacts (mortality, morbidity) or changes in the use of healthcare services such as emergency department visits was also included in our search. Only articles and reports written in English were considered, with no restriction regarding the year of publication or geographical area. Literature on climate services towards extreme weather event response planning and support other than heat events such as flood, storm, or drought, was excluded.

Participants

- Literature presenting climate services targeted toward the general population or any specific vulnerable group such as outdoor workers or the elderly.
- Humans and not animals or plants

***Concept***

Literature presenting economic assessment (Cost-benefit analysis, Cost-Effectiveness Analysis, Multi-criteria analysis)

Cost parameters could include the operational cost of climate services such as the cost of triggering heat warnings as well as the cost of response measures such as the cost of disseminating climate information to the public or the cost of healthcare professionals personally taking care of vulnerable people following heat warnings.

Literature presenting evaluation or use of climate services for heat-health adaptation in reducing health impacts (mortality, morbidity) or changes in the use of healthcare services such as emergency department visits.

***Context***

Services must be targeted to adapt to health risks posed by extreme heat events.

Services for health could be in the form of a heat-health/early warning system/app/tool, weather forecasting system/model/application, alert system.

***Exclusion criteria***

Absence of full text, review articles, a study may present an example or description of operational climate services for health but does not present an economic assessment or direct or indirect evaluation of its effect on health outcomes, climate services towards extreme weather event response planning and support other than heat events such as flood, storm, or drought.

***Search Strategy***

The search strategy aimed to locate both published and unpublished studies. WMO report on climate services for health (Shumake- Guillemot & Fernandez-Montoya, 2019) and two previously published reviews (Suckall & Soares, 2022; Toloo et al., 2013) were used as an initial study document to identify keywords to develop a full search strategy for databases: Medline (Ovid), Embase (Ovid) and Web of Science. The search strategy, including all identified keywords and index terms, was adapted for each included database as presented in Appendix 2 below. The reference list of included sources of evidence was screened for additional studies. Sources of unpublished studies/ grey literature were searched in WHO Institutional Repository for Information Sharing (IRIS), the WMO website, the Climate-ADAPT website, and Google Scholar. Since the search strategy used for databases was not adaptable to the websites, separate search strategies had to be used and are presented in Appendix 3 below.

***Selection of Evidence***

Following the search, all identified citations were collated and uploaded into EndNote, and duplicates were removed. Titles and abstracts were screened by two reviewers (PC and SS) for assessment against the inclusion criteria for the review. Some remaining duplicates were removed manually. Potentially relevant sources were retrieved in full. The full text of selected citations was assessed in detail against the inclusion criteria and exclusion criteria by the two reviewers. Any disagreements between the reviewers at each stage of the evidence selection process were resolved through discussion. The results of the search and the study inclusion process are reported and presented in the PRISMA-ScR flow diagram in Figure 1 in the main article.

***Data Extraction***

Data from selected full-text studies were extracted by two reviewers (PC and SS) independently using a data extraction sheet in Excel. Due to the nature of the varying research questions, the data extraction sheet was modified slightly while extracting data for studies answering primary or secondary research questions. Some of the common data included in the extracted sheet were: 1) Author, 2) Publication Year 2) Title/Purpose of the Study, 3) Study period, 4) Country or Region 5) Description of Climate Services 5) Cost or/and Health outcomes 6) Methods used to access cost and/or benefit 7) Sample population/vulnerable groups identified. Any disagreements that arose between the two reviewers on the data extraction process were resolved through discussion. Furthermore, when the detailed description of the climate services and response measures linked to the services were not described within the selected study and had referred to previous studies or actual service websites, those references were accessed and studied to summarize the description of the climate services for health.

***Analysis and Presentation of evidence***

A narrative summary of key findings from each article accompanying the tabulated and charted results in line with the objectives/research questions as well as some descriptions of the climate services are presented in the scoping review.

## Search string (Database)

**Database:** **Ovid MEDLINE(R) <1946 to March 10, 2023>**

**Dato:** 13,03,2023

**Total Hits:** 135

| 1 | ((monitor* or forecast* or warn* or response or detection or alert or climate or weather) adj (system$ or mechanism$ or tool$ or application* or app$ or service* or intervention or map or weather or climate or "sub-seasonal" or seasonal or "long term" or scenario or prediction or projection)).kf,tw. | 74133 |
| --- | --- | --- |
| 2 | (heat or hot or temperature$ or cold or heatwave*).ti. | 256875 |
| 3 | (mortality or morbidity or hospitali?ation or cases or incidence or evaluat* or effective* or predict or assess* or cost or costeffectiv* or costbenefit$ or "economic assessment" or "economic appraisal" or valuation or "mult-criteria analysis" or "economic evaluation$" or effect or impact).ti | 3231159 |
| 4 | (health or people or individual or "public health").kf,tw. | 3641635 |
| 4 | 1 and 2 and 3 and 4 | 135 |

**Database:** **Embase 1974 to 2023 March 10**

**Dato**: 13,03,2023

**Total Hits**: 146

| 1 | ((monitor* or forecast* or warn* or response or detection or alert or climate or weather) adj (system$ or mechanism$ or tool$ or application* or app$ or service* or intervention or map or weather or climate or "sub-seasonal" or seasonal or "long term" or scenario or prediction or projection)).kf,tw. | 96980 |
| --- | --- | --- |
| 2 | (heat or hot or temperature$ or cold or heatwave*).ti. | 267041 |
| 3 | *(mortality or morbidity or hospitali?ation or cases or incidence or evaluat* or effective* or predict or assess* or cost or costeffectiv* or costbenefit$ or "economic assessment" or "economic appraisal" or valuation or "mult-criteria analysis" or "economic evaluation$" or effect or impact).ti* | 4350042 |
| 4 | (health or people or individual or "public health").kf,tw. | 4805460 |
| 5 | 1 and 2 and 3 and 4 | 146 |

**Database:** **Web of Science Core Collection: Edition: All**

**Dato:** 21.02.2023

**Total hits:** 3554

| 1 | TS=((monitor* or forecast* or warn* or response or detection or alert or climate or weather) NEAR/0 (system$ or mechanism$ or tool$ or application* or app$ or service* or intervention or map or weather or climate or “sub-seasonal” or seasonal or “long-term” or scenario or prediction or projection)) | 965,414 |
| --- | --- | --- |
| 2 | TI = (heat or hot or temperature$ or cold or heatwave*) | 1,007,243 |
| 3 | *TI=(mortality or morbidity or hospitali?ation or cases or incidence or evaluat* or effective* or predict or assess* or cost or costeffectiv* or costbenefit$ or "economic assessment" or "economic appraisal" or valuation or "mult-criteria analysis" or "economic evaluation$" or effect or impact)* | 7,484,792 |
| 4 | TS= (health or people or individual or "public health") | 5,566,967 |
| 5 | #1 AND #2 AND #3 AND #4 | 3,426 |

## **Search string (Grey literature)**

Final Search Date: 23.02.2023

| **Website** | **Search Strategy** | **Total Hits** |
| --- | --- | --- |
| Google Scholar | ("climate service*" OR "sub-seasonal forecast*" OR "climate information" OR "warning system*" OR "weather forecast*" OR "climate forecast*" OR "daily forecast*" OR "seasonal forecast*" OR "climate projection*") AND (Heat OR temperature) | 18300 |
| Climate ADAPT | Heat OR Temperature OR warning OR forecast OR alert | 142 |
| WMO | (Heat OR temperature) AND (climate services OR Warning OR Forecast OR Alert OR Predict) AND (Cost OR benefit) AND Health | 115 |
| WHO Institutional Repository for Information Sharing | (Heat OR temperature) AND (Climate services OR Warning OR Forecast OR Alert OR Predict) AND (Cost OR benefit OR mortality OR morbidity OR Evaluation OR effectiveness)    Filters:  Language: English  Title: Climate | 251 |

## Search results

A total of 3707 records were obtained from all three databases and were exported to EndNote. Using the automatic duplicate removal function in EndNote, 178 duplicates were removed and the title and abstract of 3529 articles were screened. Based on inclusion and exclusion criteria, 77 articles were deemed relevant for full-text article screening. In the case that some articles were considered potentially useful even though the title or abstract did not exactly match the inclusion criteria, full text of such articles was studied before deciding the inclusion or exclusion and those studies are counted within the 77 articles. After reading the full-text articles, 22 articles from the database search were included in the review.

The remaining articles were excluded for a number of reasons such as some articles presented health outcomes or cost evaluation and mentioned that the effect could be due to adaptation strategy in an abstract but did not include or explicitly mention any climate services for health or HHWS as an adaptation strategy, some articles made comments on how warning systems or seasonal forecasts could help predict and prepare for heat waves to reduce health impacts without quantifying or measuring their impact, and some articles presented the effectiveness of HHWS in terms of its ability to accurately warn people about heat waves at varying thresholds and made a general comment that it could help mitigate the risk of mortality and morbidity.

In addition to the 22 articles, five articles were identified during citation searching of selected articles. Furthermore, a grey literature search was performed on Google Scholar and other organizational websites. The number of hits recorded during the search on Google Scholar was 18,300 and screening all 18,300 hits was not feasible. Therefore, hits were sorted by relevance and the first 150 hits were screened as no relevant hits were encountered even after the first 50 hits. The hits during Google Scholar and other organizational website searchers were screened first through abstracts or full text in the absence of an abstract directly from the website. Along with citation screening a total of nine studies were screened for full text. Based on inclusion and exclusion criteria a total of five studies were included. Those five articles were the ones identified through citation screening and did not include any other organizational reports or studies. Therefore, a total of 27 studies were included in the review.

## Individual results from the articles

Economic Assessment

Chiabai et al. (2018) used a cost-benefit analysis with reduced mortality as a health outcome measure. The study used three different heat wave mortality valuations to explore the main drivers influencing the economic appraisal of early heat warning systems. HWS in Madrid, Spain was used for the study. The study period over which the cost-benefit ratio was estimated was from 2020 to 2040 and Representative Concentration Pathway (RCP4.5 and RCP8.5) scenarios were used to project costs of heat alert systems, heat-related mortality, and economic benefits over the study period through economic modeling. Depending on three different mortality valuation methods used for the analysis, the study concluded the benefit-cost ratio varied from 12 to 3700.

A study by Ebi et al. (2004) estimated the economic benefit of reducing heat-related mortality and the number of lives saved after the implementation of the heat watch/warning system in Philadelphia. This study showed that issuing an individual warning saved about 2.6 lives on average which was calculated to be around 117 lives saved during the period 1995-1998. Valuing the number of lives saved produced a gross benefit of about $468 million over the 3-year period, and the cost of implementing the warning system in Philadelphia as compared to the benefit was reported negligible.

Hunt et al. (2017) conducted a cost-benefit analysis of a heatwave warning system implemented in three major European cities over a 50-year period between 2015-2064 using future climate change scenarios and economic modeling. Economic appraisals of the three systems in three different cities/countries were analyzed separately. Heat-related mortality and heat-related patient days were used as health outcomes in the study. The analysis showed that the adaptation cost might increase with climate change scenario but the increase in benefit outweighs the cost increase. The benefit-cost ratio under various climate change scenarios for all three cities remained positive in the main analysis and varied between 11 and 1880. However, sensitivity analysis by assuming reduced effectiveness of HWWS and lower bound value of mortality and per patient days found in the existing evidence concluded a significantly lower benefit-cost ratio in two cities and a negative benefit-cost ratio in one city. The negative benefit-cost ratio implied that, in that particular sensitivity analysis scenario, the cost-effectiveness of HWWS did not exist anymore.

Williams et al. (2022) conducted a cost-benefit analysis of the South Australian heat health warning system. Unlike the above 3 studies with mortality as health outcomes, the authors used reductions in hospital admissions and ambulance callouts as a measure to value the benefits of the system. The study concluded with an estimated benefit-cost ratio between 2.0 and 3.3.

Cost of Heat Warning System

Chiabai et al. (2018) categorized the cost of a heat warning system into two categories: basic intervention and basic intervention plus supplementary action. The basic intervention included the everyday cost of additional labor required to maintain emergency medical services and a heat line while supplementary action included costs of a wider set of actions such as media announcements, dissemination campaigns, and community outreach programs that help provide extra care to heatwave vulnerable groups and alerts to nursing homes. The cost per heatwave day for basic intervention was reported to be 7,800 Euros and for basic intervention along with supplementary action was reported to be 14,000 Euros.

Similarly, the cost of HHWS estimated in the study by Ebi et al., 2004 included some direct costs such as wages of Heat line and additional Emergency Medical Service (EMS) crews. While the cost of other measures was not described completely, a total of 10,000 US dollars per heat wave warning was used for the analysis. The study by Hunt et al., 2017 also included the wages of health professionals as an estimate of the cost of HHWS. Apart from wages, the study estimated an additional fixed annual cost of 200,000 Euros which included the annual contract fee of the weather office and warning dissemination costs.

The cost of HHWS was comprehensively described in the study by Williams et al. (2022). The study included the cost of pre-seasonal activities, seasonal activities, warning activation, and post-activation activities. A total cost of 593,000 Australian Dollars was estimated for implementing the HHWS and targeted intervention for seven days. Mortality:Benmarhnia et al. (2016) conducted a study to calculate the impacts of early heat alerts and advisories combined with emergency public health measures (collectively termed a heat action plan, HAP) on health-related mortality in Montreal, Quebec. Using a difference-in-differences approach, the study concluded that HAP reduced mortality during hot days by 2.52 deaths per day. The estimated benefit of HAP was even more significant among the elderly and individuals living in neighborhoods with low socioeconomic status as the difference in the mortality rate among these groups as compared to non-elderly and individuals living in neighborhoods with high socioeconomic status during the post-HAP period was calculated to be lower.

## Chau et al. (2009) assessed the relationship between a very hot weather warning (VHWW) and mortality due to stroke and ischemic heart disease (IHD) among the elderly (≥65 years old) in Hong Kong during the summers of 1997-2005 using multiple linear regression. A VHWW was a reminder for the public to take preventive measures and specifically for the relevant government departments to take necessary actions. The study concluded that the average mortality due to IHD and stroke on the days with VHWW was less than on the days without VHWW. The study also found that 19% of deaths from IHD and 14% of deaths from stroke could have been reduced in 1997-1999 if VHWW was implemented.

## de ‘Donato et al. (2018) assessed the change in the impact of heat on mortality in Italy after the implementation of the national heat plan. One of the main components of the Italian HAP is a city specific HHWS. The association between temperature and mortality before and after the implementation of the national heat plan was studied using the distributed lag non-linear models (DLNM). The study predicted that around 1900 total heat-related deaths were averted between 2005 and 2016 after the implementation of the HAP.

## A study by Fouillet et al. (2008) assessed the health impact of the French HHWS along with public preventive measures and general awareness due to previous heat waves. The expected number of deaths during 2004-2006 was estimated based on the temperature and mortality relationship from 1975 to 2003 using the generalized estimating equations (GEE) approach. The authors reported that observed deaths in 2004 and 2005 were lower than the predicted deaths by 2-8%. In 2006, based on the previous heat-mortality relationship, 6452 excess deaths were predicted, while there were about 2065 observed excess death. This substantial estimated mortality deficit of 4388 deaths was partially attributed to the set-up and implementation of the HHWS.

## Heo et al. (2019) estimated the effect of implementing a heatwave warning system in reducing mortality risk in Korea using a difference-in-differences approach. The authors did not find a significant decrease in all-cause mortality in the all-age group as an effect of heat warnings. However, they found a significant decrease in cardiovascular mortality due to the warning systems in unemployed elderly (age 75+), widowed elderly (age 75+), young adults (age 19-64) with no education, and elderly (aged 65+) with university or higher degrees. Similarly, they found a significant decrease in respiratory mortality due to the warning systems in children and youths (age 0-19), widowed and single elderly (age 65+), uneducated young adults (age 19-64), elderly with elementary education (65+) and elderly with a university education or more degrees (75+).

## A study conducted by Hess et al. (2018) assessed the impact of HAP on all-cause mortality in 2007-2010 (pre-HAP) and 2014-2015 (post-HAP) in Ahmedabad, India. A HHWS is used to trigger public health responses within HAP. A DLNM was used to assess the relationship between temperature and mortality before and after the introduction of HAP. The authors observed a reduction in all-cause mortality on all warning days after HAP implementation. Annually about 1190 deaths were estimated to be avoided in the post-HAP period.

## Heudorf and Schade (2014) measured the effect of a HHAP and heat warning system on mortality in Hesse, Germany. The authors compared the mortality and metrological data using the Kruskal-Wallis test and Mann-Whitney test. The heat warning system was implemented in 2006, and the effect on mortality from 2004-2013 was compared to 2003. There was a significant increase in mortality rates during the heat wave in 2003 compared to the average daily mortality rates in the summer from 2004-2013. The excess mortality rate during that period was 78% for the entire population, 113% for those aged over 80 years, and 64% for those aged 60 to 79 years. However, during subsequent heat waves in July 2006, 2010, and 2013, the excess mortality was reduced to a maximum of 23%, 12%, and 4% of overall excess mortality in 2010, 2006, and 2013, respectively. These reductions in excess mortality were partly attributed to the implementation of a heat early warning system.

## A study by Martinez-Solanas and Basagaña (2019a) assessed the impact of actions taken in the regional Spanish heat health preventive plan (HHPP) on the health effect due to extreme heat with a slightly different Period 1 than the previous study from 1993-2002. The HHPP uses a heat alert system to activate public health measures. The relationship between temperature and mortality was estimated using time-varying distributed log non-linear models. The authors found a decrease in heat-related mortality during extreme heat in the second period with a greater reduction in provinces that employed more actions from HHPP. The elderly population in the smaller municipalities experienced more notable reductions in mortality due to heat during the second time period. There was a minor decrease in mortality attributed to extreme heat in the second period (0.56%) than in Period 1 (0.67%). The decrease was most notable among older adults, deaths related to cardiovascular issues, and in towns with high socioeconomic vulnerability. However, during the second period, there was an increase in mortality during moderate heat.

## Michelozzi et al. (2006) conducted a study to explore the temperature-mortality curve to assess temporal and geographical variations in 4 cities in Italy. A segmented regression and generalized additive models were used to explore the relationship between mortality and maximum apparent temperature. A HHWS was established in 2003 in Italy. The study reported that for the same exposure interval, the impact of temperature on mortality was lower in 2004 than in 2003 in some cities. The authors suggested that the reduced mortality could be due to the implementation of public health interventions supported by warning systems along with the lower average and cumulative level of exposure and a less vulnerable population left after the 2003 heat wave.

## Another study in Italy by Morabito et al. (2012) conducted a study to measure temporal modification of heat-related mortality in elderly (≥75 years) and older adults (65-74 years) after the implementation of a HHWS in Florentine area. The HHWS was implemented in 2004. The study uses case-crossover time-stratified designs. The result showed that the variation in heat-related mortality odds ratio (OR) was non-significant between the pre-intervention and post-intervention periods. However, the mortality OR among people aged 75 and over gradually decreased from 1.23 during 1999-2002 (pre-intervention), to 1.12 during 2004-2005 (experimental HHWS only for Florence) and to 1.12 during 2006-2007 (official HHWS extended to the whole of Florence area). The author suggested that HHWS could have contributed significantly to reducing the number of days needed to overcome heat-related mortality.

## A study conducted by Palecki et al. (2001) aimed to evaluate whether the actions taken after the 1995 heat wave in Chicago and St. Loius contributed to a better response to the 1999 heat wave and if there were additional insights to be gained from the 1999 event. Heatwave responses included a heatwave warning and Hot Weather Health Advisory (HWHA). This was a retrospective observational study. After studying the response efforts for the heat waves in Chicago and St. Louis, the authors found that both cities were successful in reducing the number of deaths caused by heat waves. In St. Louis, the 1999 heat wave was intense for a much longer duration and resulted in 36 deaths while only 27 died during the 1995 heat waves. On the other hand, Chicago experienced a sharp decrease in the number of deaths during heat waves from 700 in 1995 to 114 in 1999. However, in the 1999 heat wave, the mortality rate was almost equal in both cities, at around 1.4 per 100,000 people. The author concluded that since the mortality rate was similar in both metropolitan areas, both cities had an effective heat emergency response system and partly attributed the reduction in the number of deaths in Chicago to the heatwave warnings and response measures following the warning.

## Ragetti et al. (2017) measured the impact of heat on daily mortality in 8 Swiss cities during 1995-2002 and 2004-2013, the latter time period characterized by the implementation of public health intervention to protect against extreme heat. Heat warnings were one of the interventions that provided alerts for health professionals and care for vulnerable individuals during heat waves. The temperature-mortality relationship was assessed using quasi-Poisson regression models with non-linear distributed lag functions. The authors found a decrease in relative risk of mortality estimates for the entire population with the risk ratio being 1.13 before 2003 and 1.09 after 2003. However, this change was not significant. Nevertheless, the authors reported that the decrease in risk ratio during high summer temperatures was mostly attributable to three cities with heat warning systems.

## Schifano et al. (2012) assessed how heat affected mortality rates in the elderly over 65 years old, both before (1998-2002) and after (2006-2010) the introduction of the national heat health prevention program in Italy. The study included 16 cities in Italy with active HHWS. The study showed that after the introduction of the Italian national prevention plan, there was a notable reduction in the impact of high temperatures on the mortality rate of individuals aged 65 years and above. However, some variations were observed among different cities. The reduction was observed only in cases of high temperatures whereas no changes or increases in the impact on mortality were detected with lower temperatures.

## Steul et al. (2018) conducted a study to examine the possible impact of the heat-health action plan for Hesse (HHAP) including HHWS, which was implemented after the 2003 heatwave, by comparing the mortality during the 2003 heatwave with the heatwave mortality during subsequent years till 2015. The relationship between maximum apparent temperature and mortality was assessed using a Generalized Additive Model (GAM) model. The result showed that even though the mortality rate in 2006, 2010, and 2015 was lower than the 2003 mortality rate, the authors concluded the effectiveness of the heat warning system and action plan was inconclusive due to varying characteristics of heatwaves during the pre-HHAP and post-HHAP period.

## One of the objectives of the study carried out by Tan et al. (2007) was to examine whether varying socioeconomic factors in Shanghai can explain some of the daily mortality differences during heat waves in 1998 and 2003. The author found that even though the heatwaves during 2003 were longer-lasting as compared to the summer of 1998, fewer mortality was observed. The author partly attributed these lower mortality rates to the implementation of a heat/health watch warning system in 2002.

## Another study by Weinberger et al. (2018) measured the variation in mortality rates associated with heat alerts issued by NWS in 20 cities in the United States during the period 2001 to 2006. The study found that in general, heat alerts were not linked to reduced mortality rates. Only in Philadelphia, heat alerts were related to a decrease in mortality rate of 4.4%. Assuming such reducing in mortality to be causal, the authors reported that heat alerts averted around 45.1 deaths annually between 2001 and 2006. However, there was no statistically significant association between heat alerts and reduced mortality rate observed in other cities.

##

## Morbidity and Healthcare Services Utilization

## A study by Benmarhnia et al. (2019) measured the change in hospital admissions for heat-related risk among Medicare fee-for-service beneficiaries (aged 65 years or older) in New York City, as the effect of change in threshold to activate heat emergency plan. The threshold to trigger heat advisories was lowered in 2008 based on local epidemiological studies. The study reported that on average 0.80 fewer heat-related illnesses per day were observed during the hot days in 2009 and 2010 which was equivalent to preventing approximately 50 heat-related illnesses over the 2 years.

## Clemens et al. (2022) evaluated the effects of harmonized heat warning and information system (HWIS) on emergency department visits (ED) for heat-related illnesses in Ontario, Canada. The study did not find any significant decrease in the rates of ED visits for any subgroups or the general population. In contrast, after the intervention, individuals with a recent history of homelessness had an increase in the rate of ED visits of 3.07 (95% CI 0.63-5.51, p= 0.02) which was statistically significant.

## A study by Martinez-Solanas and Basagaña (2019b) measured the effects of heat on hospital admissions in Spain during two time periods 1997-2002 and 2004-2013, the second one characterized by the introduction of SHHPP which is activated using a heat alert system. The authors found that after the introduction of the heat health prevention plan, the effect of heat on all-cause hospital admission slightly reduced while excluding 2003 because 2003 was an outlier with exceptionally high temperatures. Heat-related hospitalization for respiratory diseases was slightly reduced in the second period. During period 1, there would have been a small and statistically insignificant increase in the risk of hospitalization related to cardiovascular diseases on days when the HHPP would have been activated. However, during period 2, the activation of the plan showed a small protective effect for hospitalizations related to cardiovascular diseases.

## A study by Mehiriz et al. (2018) assessed the effect of an automated phone heat warning system, Téléphone Santé, on health service use and health behavior through a randomized controlled study. The health service uses included call or consultation with healthcare professionals such as doctor, nurse, or pharmacist, call to health information services, and emergency department visit. The result showed that there were no significant differences observed between the treatment and control groups regarding the occurrence of heat-related symptoms. In the treatment group, although insignificant, fewer participants (7.7%) used the healthcare system compared to those in the control group (9.4%). However, women in the experimental group used the healthcare system less than the women in the control group (5.7% versus 11.3%). This difference between the two groups was significantly greater for women with chronic illnesses (6.2% versus 13.5), which amounted to a 54% difference. Thus, the phone heat warnings reduced health services usage among women, especially those with chronic illnesses related to heat vulnerability.

## Mortality and Morbidity

## Nitschke et al. (2016) assessed the impact of a heat warning system on morbidity and mortality in Adelaide, South Australia. The extreme events of 2009 (pre-intervention) were compared to those of 2014 (post-intervention) in a case series analysis. The analysis concluded that there were fewer ambulance callouts, hospital admissions, and emergency presentations during the heatwave events in 2014 as compared to 2009. There were 297 (9%) fewer total ambulance callouts and 207 (59%) fewer cardiac-related callouts in 2014. Furthermore, an estimated 134 (30%) fewer renal cases were observed in 2014.

## A study by Weinberger et al. (2021) determined the association between heat alerts and cause-specific hospitalization and all-cause mortality among Medicare beneficiaries aged 65 years and above. The study covered 2,817 counties, and the data was collected between 2006 and 2016. The authors reported that heat alerts did not reduce the risk of mortality but were associated with an increased risk of hospitalization for heat stroke, and fluid and electrolyte disorders.

## Weisskopf et al. (2002) investigated whether the decrease in heat-related deaths and paramedic runs in 1999 was solely due to variations in heat levels. The study took place in Milwaukee County, USA, and compared data from 1995 to 1999. The study reported fewer heat-related mortality in 1999 than in 1995. The observed death rate and Emergency Medical Services (EMS) runs during heat advisory days in 1999 were less than the predicted rate made using the 1995 relation between heat and heat-related death or EMS runs. The authors partially attributed this reduction to heat advisory prior to heatwaves.

## S2: Interview methodology and results

An interview guide was developed comprising 10 questions to investigate how the climate services were developed and which stakeholders they target, how the services are financed, measures implemented to promote capacity-building among target users and to involve vulnerable groups, communication strategies, steps taken to evaluate the impact and use of the services, and barriers encountered in the development and implementation of the services. The interviews were moderated by two researchers from the Norwegian Institute of Public Health, using Microsoft Teams. The interviews lasted 40 minutes on average, were video recorded, transcribed with verbatim, and analyzed in Microsoft Word using a simple content analysis. Text pertaining to each question in the interview guide was reviewed and organized in overarching coding categories. Then, the text was summarized and broken down into smaller sections, focusing on the most important points, and relevant quotes were identified. Points that contained similar ideas were merged and gathered in subcategories of coding. Finally, the interview data and the summary data were reviewed once more to ensure that the data were presented in an accurate manner and no essential information was missing.

The interview guide was slightly modified to conduct the interviews with the public health representatives to make the questions relevant to them, although the content of the questions remained unchanged. The representatives were contacted by email using the network of the ENBEL project [ref] and those who accepted our invitation helped us recruit other interviewees. The representatives in our sample signed a written informed consent form (Appendix 4). The interview study was reviewed by the Data Protection Officer of the Norwegian Institute of Public Health and assessed as not requiring ethics approval by an ethics committee.

Table S2: Interview Questions

| Technical aspects | 1. About the tool  i. Shortly describe the tool [you are using/contributing to develop] and its status.  ii. Which data feeds the tool [are you using] (e.g., climate data, health data)?  2. [If involved in tool development]: End-users and Stakeholders:  i. Who are the end-users of the service?  ii. Who are the stakeholders in the development of the service?  3. [If involved in tool development]: How is the service financed and your views on its sustainability? |
| --- | --- |
| Social aspects | 4. [If involved in tool development], or [“Were you trained in the use of the tool?”] Were measures implemented to promote capacity-building among target users (E.g, training community leaders, volunteers and health workers in the use and management of the service, building up data management capacity and IT-infrastructure, developing outreach materials targeting high-risk groups)  5. [If involved in tool development], or [“Do you know if…?”]: Does the tool identify and target any specific vulnerable groups? If yes, how was consideration of the needs, risk perceptions, behaviors, priorities, and practices of vulnerable groups integrated in the design of the service?  6. [If involved in tool development]: Were representatives from targeted users as well as vulnerable and high-risk groups involved in the design of the service? E.g., representatives from groups such as migrants, seniors, CVD patients, outdoor workers, pregnant women, geographically isolated groups? If yes, how? E.g., workshops, surveys  7. [If involved in tool development]: Which communication strategies were implemented to inform users, and vulnerable and high-risk groups about the service? (E.g, partnering with the media, use of social media, text messages, web portals, multilingual information, infographics, reducing jargon) |
| Evaluation | 8. [If user]: What is your experience og using the tool? How are you using it? Identified benefits (how were they identified)? Anything you miss?  9. [If involved in tool development] or [Have you evaluated the use of the tool?]: Has the use of the service been evaluated? If yes, how? E.g., evaluation meetings, surveys, number of saved lives, increased awareness of climate-related health risks.  10. How would you describe the service’s collective impact?  11. [If involved in tool development]: Which barriers did you encounter in the development and implementation of the service? E.g., risk warning information not understood or not trusted and how were/are these barriers addressed?  12. [If user]: What are your plans for future use of the tool? |

Table S22: Heat-health climate services and public health authorities included in the interviews

| **Service** | **Country** | **Organization** | **Description** | **Number of representatives (N=11)** | **Role or representative(s)** | **Interview date** |
| --- | --- | --- | --- | --- | --- | --- |
| [ClimApp](https://www.lth.se/climapp/about-climapp/) | EU (partially operational*) | ClimApp project | App | 1 | Project lead | 17/02/2023 |
| [European Health Service](https://climate.copernicus.eu/european-health-service) | EU | Vito (Flemish research organization) | Web service | 1 | Product developer | 03/03/2023 |
| [Heatshield](https://www.ncbi.nlm.nih.gov/pmc/articles/PMC6718993/) | EU (Discontinued) | Heat-Shield project | Web service | 1 | Project lead | 04/03/2023 |
| [Copernicus Climate Change Service](https://climate.copernicus.eu/) | EU | European Centre for Medium-Range Weather Forecasts | Web service | 1 | Project lead | 10/03/2023 |
| [Italian Heat Health Watch Warning system](https://www.deplazio.net/en/national-system-hhww) | Italy | Lazio Regional Health Service | Web service /app | 1 | Researcher | 22/03/2023 |
| [Heat Health Warning System- Germany](https://www.mdpi.com/2073-4433/11/2/170/htm) | Germany | University of Freiburg | Web service/app | 1 | Researcher | 31/03/2023 |
| [South African Weather Services](https://www.weathersa.co.za/) | South Africa** | South African Weather Services | Web service | 1 | Researcher | 17/04/2023 |
| Santé Publique France | France | Santé Publique France | Public Health Agency | 1 | Project lead | 27/12/2023 |
| UK Health Security Agency | United Kingdom | UK Health Security Agency | Public Health Agency | 3 | Team lead /researchers | 5/12/2023 |

*Operational without anyone taking in charge of updating the system, **Due to resource constrains only one stakeholder from Africa was included for this study

Table S23: Short description of the climate services include in the interview

| **ClimApp:** <https://www.lth.se/climapp/about-climapp/> |
| --- |
| ClimApp is a multilingual application developed by the EU-project ClimApp funded by the European Research Area for Climate Services (ERA4CS) and three participating countries: Sweden, Denmark, and the Netherlands. The app integrates weather forecast data into human heat balance models and heat stress indices. It uses short-term weather forecasts such as air temperature, humidity, air velocity, and solar radiation and individual factors such as acclimatization to heat, activity intensity, and clothing that can affect the body heat exchange and health.  The app predicts body responses and provides personalized health risk warnings and advice for individuals, public and private sectors, to support decision-making to cope with heat and cold stress when facing extreme weather events such as heat waves and cold spells. The app is automatically connected to the local weather forecast and provides a forecast for 24 hours. It was officially launched in the summer of 2019. |
| **The European Health Service:** <https://climate.copernicus.eu/european-health-service> |
| The European Health Service, as a part of the Copernicus Climate Change Service (C3S), provides users with tailor-made climate information for the health domain. This information consists of climate-health indicators concerning heat and cold stress, vector-borne diseases, and allergenic pollen. The service includes [data sets and applications](https://climate.copernicus.eu/european-health-service) and some are integrated into the services of the European Environment Agency and the European Climate and Health Observatory. The applications provide information for all regions and countries in Europe. The service collects heat data from multiple countries and produces mortality impact estimates for several cities.  The allergenic pollen application estimates how climate change impacts the onset of the pollen season for specific pollen and the length of the pollen season using seasonal forecasts. Another application calculates the suitability of the tiger mosquito (survival) at regional and urban scales using temperature and precipitation estimates. |
| **Copernicus Climate Change Service (C3S):** <https://climate.copernicus.eu/> |
| C3S is implemented by the European Centre for Medium-Range Weather Forecasts (ECMWF) on behalf of the European Commission. C3S provides environmental and climate information data and tools to the European Climate and Health Observatory and the European Environment Agency. C3S is one of [six thematic information service](https://www.copernicus.eu/en/services)[s](https://www.copernicus.eu/en/about-copernicus) and provides information climate data and information on impacts on a range of topics and sectoral areas through a Climate Data Store (CDS). C3S develop apps that can be used by the general public, e.g., pollen spreading forecast, using AI. The data and the apps are freely available and although the focus is on Europe, some data have global coverage. |
| **HEAT-SHIELD:** <https://heatshield.zonalab.it/> |
| HEAT-SHIELD was a Horizon 2020 project (EU funded - grant agreement No 668786; see [www.Heat-Shield.eu](https://eur04.safelinks.protection.outlook.com/?url=http%3A%2F%2Fwww.heat-shield.eu%2F&data=05%7C01%7CIsabelle.Budin.Ljosne%40fhi.no%7Ca84d1ff08fa242dde54b08db5ced9840%7C54475f801baa4ea99185c0de5cc603fe%7C0%7C0%7C638205947764369565%7CUnknown%7CTWFpbGZsb3d8eyJWIjoiMC4wLjAwMDAiLCJQIjoiV2luMzIiLCJBTiI6Ik1haWwiLCJXVCI6Mn0%3D%7C3000%7C%7C%7C&sdata=B68cylRnBmivSLFfjDsi4Y8%2Fuyet8HwxSN6%2FC%2B3Ce1Q%3D&reserved=0) for details) that developed a multilingual occupational warning system platform operating for Europe. It provided forecasts for daily maximum heat stress in Europe. The system also provided information about personalized local heat-stress-risk based on workers' physical, clothing, and behavioral characteristics and the work environment (outdoors in the sun or shade), factors such as temperature, airspeed, radiation, and wind and also taking into account heat acclimatization. The service calculates estimates and produces warning levels from green (no expected heat stress), to yellow and up to red. It provides behavioral suggestions (hydration and work breaks recommended) to be taken into consideration in the short term (5 days) together with long-term heat risk forecasts (Morabito et al., 2019). The service was primarily developed as a concept and is currently not operational, although some warnings may be available to already-registered users. |
| **The Italian Heat Health Watch Warning System (HHWW Systems):** [**https://www.deplazio.net/en/national-system-hhww**](https://www.deplazio.net/en/national-system-hhww) |
| The Italian Heat Health Watch Warning system was developed for urban areas where heat-related health effects are well-known, and response needs to be intensified. The system, operational since 2004, offers 3-day warning systems for 27 major cities (regional capitals and cities with more than 250.000 inhabitants) based on temperature-mortality association and the identification of air masses most at risk for health. It considers the population decrease throughout summer and adaptation to temperatures throughout summer. The system issues a graded warning bulletin during the summer period with four levels of warning: from 0 (no risk) to 3 (heat wave persistent high risk for 3 or more consecutive days). Level 1 is the pre-alert level aiming to give stakeholders time to activate emergency protocols. The system also provides recommendations and guidance at the national level on behalf of the Ministry of Health for specific vulnerable groups (elderly, people with chronic disease, children, workers, pregnant women) and entails the formal identification of elderly vulnerable subgroups which are actively monitored (home visits, phone calls, tele-medicine, and e-health services etc.) throughout the summer by the general practitioners, health, and social services based on local plans in place. |
| **The German Heat Health Warning System (HHWS):** <https://www.mdpi.com/2073-4433/11/2/170/htm> |
| The German Heat Health Warning System has been running since 2005 and provides heat warnings at the county level. It uses a combination of meteorological and epidemiological data and calculates the perceived temperature to assess the levels of heat stress and produce warnings. The forecast system further comprises the nocturnal indoor conditions, the specific characteristics of the elderly population, the urban heat island effect, and the elevation of a region. In addition, information about UV, weather sensitivity, and pollen spread about heat trends for up to 6 days based on perceived temperature and indoor conditions. The warnings are available on a website and smartphone apps. |
| **The South African Weather Services:** <https://www.weathersa.co.za/> |
| The South African Weather Services provide impact-based weather forecasts and information about the probable impact of temperature on people since 2015.  Several heat-related products are issued to the public including the heatwave advisory. In many instances, SAWS will issue a media release to internal personnel and external stakeholders using multichannel media platforms. A discomfort product is also issued to the public, with no advisory band but, feel-like temperature values, that incorporate the humidity. The systems provide a discomfort forecast of 72 hours sent out to the public and one forecast predicting the percentage of people who will be impacted at specific times and should take precautionary measures, e.g., stay under shade, drink water, and wear light clothing. The main stakeholders are the sectors of health care, agriculture, and energy. |

**Table S24: Selected Quotes from interviews**

**Climate services and vulnerable groups**

*“You might target everybody, but you do this much more for vulnerable people because you know they are the ones who are the most sensitive. For example, pollen. If you just need to take a pill, you might not make the effort to have an application on your phone. But if you know that if you will have an asthma crisis after walking 200 meters, you may be putting your life in danger, using the service will be important. “*

*“We need to be cognizant that we should not be, you know, sitting in our offices and developing all these wonderful solutions because at the end of the day, they're not going to be used by us, they will be used by the people. If you involve the people, then they take ownership of whatever product we are developing for them (…). For me, it's about complementing the scientific information with the information that is sitting with the communities.”*

***Data collection, reliability and sharing***

“*Keeping it simple is key. There is no point having too many variables in it because it does end up causing more errors or it's harder to understand. Even apparent temperature can be hard to make understandable.”*

*One of the things which is difficult is to provide advice in terms of hydration. We know that one worker may sweat half a liter and another one and half a liter. Even though we developed some biophysical models based on the input parameters and the clothing and the individual metabolic information, it's still very difficult to say whether this worker should drink one liter, should or shouldn't add salt to meals and drinks and so on, because of individual differences”.*

**Communication strategies to inform users, and vulnerable and high-risk groups about the services**

*“The next heatwave will come, and we must be prepared. One thing I always do during my presentations about the heat health warning system, every 5 minutes, I make a stop and say, sorry, and I drink. We need easy, understandable examples, and this must be provided, that's the point.”*

*“One size fit all approach like normal weather forecast does not work well for both fit and healthy people and vulnerable groups”* and that *"personalization" is one further step/approach to take into account vulnerability”*

**Views on the climate services’ collective impact**

*“One important thing is that we do not aim to reduce mortality. We want to reduce the exposure. (…) Reducing mortality is not the appropriate question (…). Mortality is the final stage, people will not be alive again, they will not recover, you know.”*

***Interdisciplinary work***

*“Something that looks obvious for us is not for others [working in health]. For instance, temperature. We can have different ways to compute temperature based on different maps, different backgrounds, different analytical ways and for us is makes sense to have several different ground temperatures. It doesn't always make sense for others, and we need to find a way to express better ourselves to make it sensible to others.*

***Awareness raising, communication and dissemination.***

*“We, as scientists, want people to be prepared for the heat wave that will hit them. We want them to prepare now. From the company side, some of them know that already and they take some precautions, but the workers often don't get aware of it until the heat is there. And then it's too late for preparedness. (…) The heat stress and cold stress for that matter, is like out of sight, out of mind.”*

*“Not all vulnerable groups know they are vulnerable. Some think … well, I'm not at risk, you know, but yes, you might be. And that also means young people, athletes training at midday, for example, they're not generally at risk, but if they train during heat waves, they might be at risk as well, so really getting them to (…) know what dehydration means, what the symptoms, what to do, for instance, stay in a cool room for half an hour or drink a lot, is relevant.”*

*“People coming to the hospital often already have chronic several chronic conditions, they might have diabetes, they might have COPD, they might have something else. And it's easier to focus on the worsening of that and not really relate it to heat and dehydration.*

## S25: Participant information and consent form


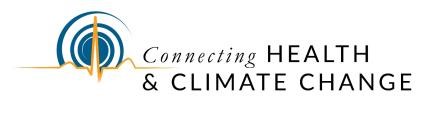


DO YOU WANT TO PARTICIPATE IN THE RESEARCH PROJECT ENBEL?

We would like to invite you to participate in a research project on strategies for climate and health adaptation.

THE PURPOSE OF THE PROJECT AND WHY YOU ARE BEING ASKED

The purpose of the ENBEL project (www.enbel-project.eu/) is to support the formulation of European climate and health guidelines by obtaining and summarizing new knowledge about climate adaptation measures. ENBEL brings together international research projects funded under the Belmont Forum's Collaborative Research Action (CRA) in the EU's Horizon 2020. As a deliverable within ENBEL project, this research aims to identify costs, benefits, equity, and social implication of climate services for heath used as an adaptation strategy. You are invited to participate in the ENBEL project because you have been identified as a key stakeholder working with climate services for health as an adaptation strategy.

WHAT DOES THE PROJECT MEAN FOR YOU?

You are invited to participate in a single interview of approximately 60 minutes to discuss the technical and social aspects, and the evaluation of the climate services you are involved with. The interview will be conducted digitally and will be recorded and transcribed. All results will be completely anonymized and will not be linked to you. Your participation is voluntary, and you can withdraw from the project at any time.

We will register the following information about you: name, surname, email address and phone number. The interview data will be processed without name or other directly identifiable information (= encoded data). A code connects you to your data through a list of names. Audio recordings and transcription files will be stored on a secure server at the Norwegian Institute of Public Health. The interview data and personal information can only be used by the project team. Any personal data, including audio recordings, will be deleted after publication of the results and no later than 30 April 2024. The information you provide will only be used in the ENBEL project and no other projects.

POSSIBLE ADVANTAGES AND DISADVANTAGES

We are not aware of any risks or drawbacks of participating in this research.


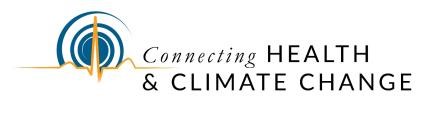


VOLUNTARY PARTICIPATION AND THE POSSIBILITY TO WITHDRAW YOUR

CONSENT

Participation in the project is voluntary. If you wish to participate, please sign the declaration of consent on the last page. You may withdraw your consent at any time and without giving any reason. It will not have any negative consequences for you if you do not want to participate or later choose to withdraw. If you withdraw your consent, no further research will be conducted on your information. You can demand access to the information stored about you, and these will then be disclosed within 30 days. You can also demand that your information in the project be deleted. The right to demand destruction, deletion or disclosure does not apply if the material or information is anonymized or published. If you later wish to withdraw or have questions about the project, please contact Shilpa Rao, Senior Researcher (see contact information below).

WHAT HAPPENS TO THE INFORMATION ABOUT YOU?

The information registered about you will only be used as described above and is planned to be used until 30 April 2024. You have the right to access what information is registered about you and the right to have any errors in the information registered corrected. You also have the right to access the security measures when processing the data. You can complain about the processing of your information to the Norwegian Data Protection Authority and the data protection officer (see contact information below)

CONTACT INFORMATION

If you have any questions about the project or wish to withdraw from participation, please contact Ms. Shilpa Rao, Senior Researcher, Phone: 21 07 84 05, shilpa.rao@fhi.no

If you have any questions about data protection in the project, you can contact the data protection officer at the Norwegian Institute of Public Health: personvernombud@fhi.no

TO THAT MY PERSON BIOLOGICAL MAT

I agree to participate in the project.


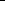

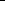

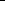

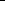

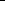

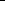

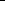

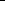

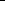

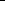

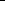

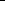

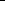

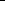

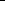

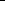

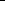

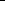

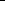

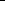

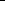

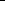

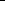

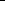

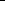

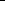

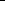

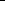

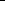

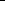

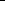

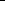

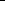

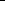

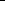

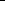

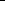

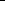

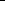

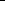

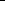

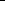

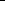

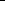

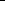

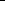

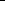

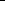

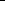

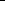

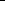

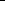

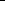

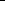

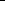

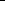

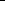

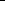

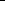

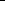

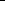

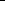

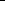

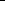

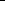

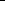

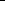

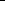

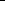

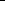

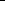

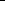

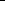

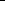

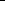

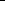

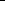

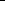

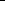

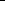

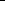

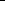

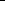

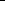

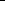

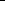

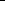

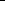

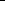

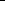

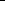

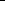

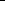

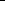

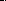

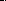

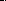


| Place and date | Participant’s signature |
| --- | --- |
|  |  |

Participant's name in printed letters
