# Supplementary figures and images for: Evaluating the socioeconomic benefits of heat-health warning systems
Source: Eur J Public Health. 2025 Feb 9;35(1):178–86. doi: 10.1093/eurpub/ckae203 (PMC11832133; doi:10.1093/eurpub/ckae203)

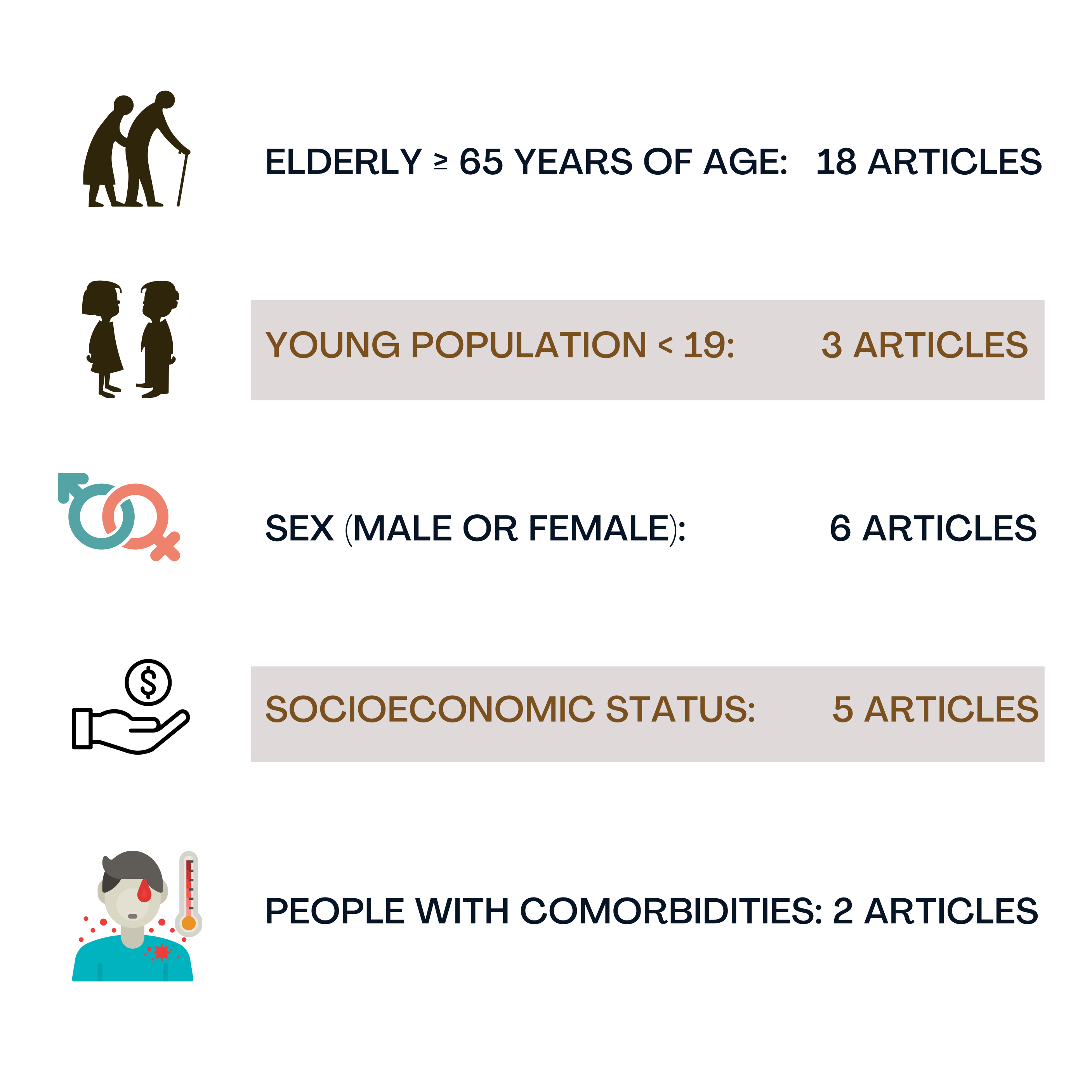

Supplement: ckae203_Supplementary_Data [file ckae203_supplementary_data.zip › ckae203_Supplementary_Data/ejph-2024-05-om-0342-File007.tiff]

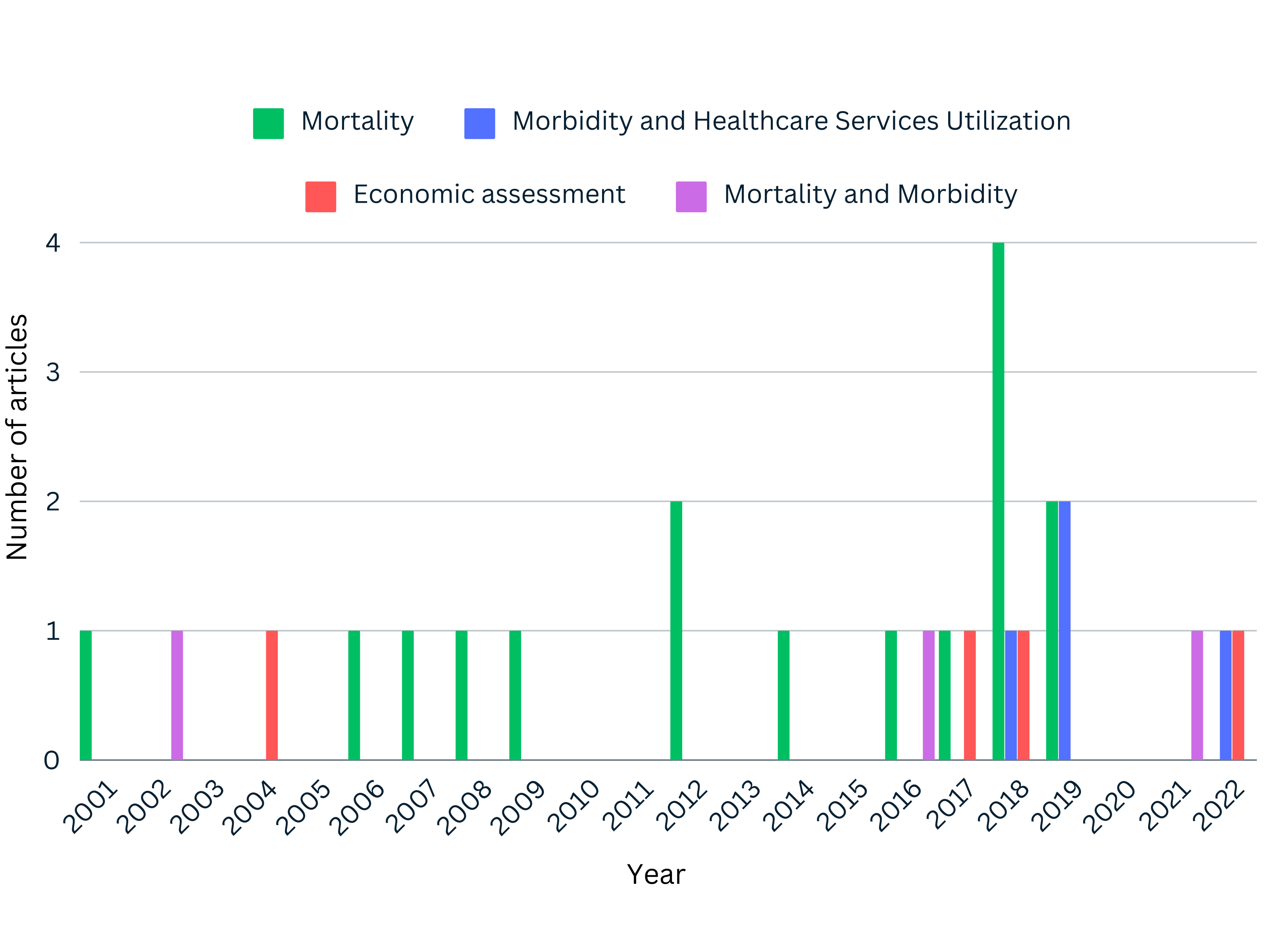

Supplement: ckae203_Supplementary_Data [file ckae203_supplementary_data.zip › ckae203_Supplementary_Data/ejph-2024-05-om-0342-File008.tiff]

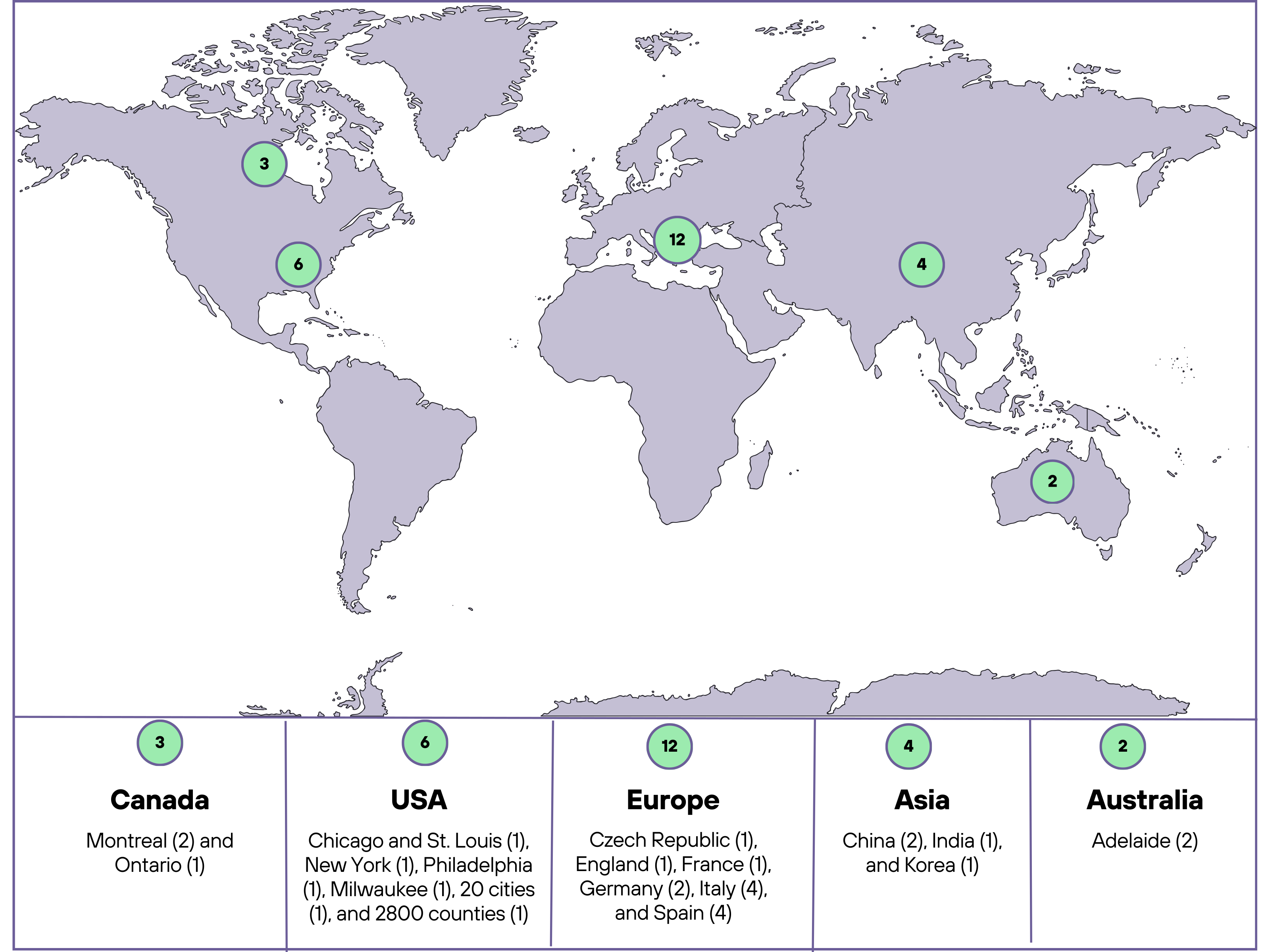

Supplement: ckae203_Supplementary_Data [file ckae203_supplementary_data.zip › ckae203_Supplementary_Data/ejph-2024-05-om-0342-File009.tiff]
